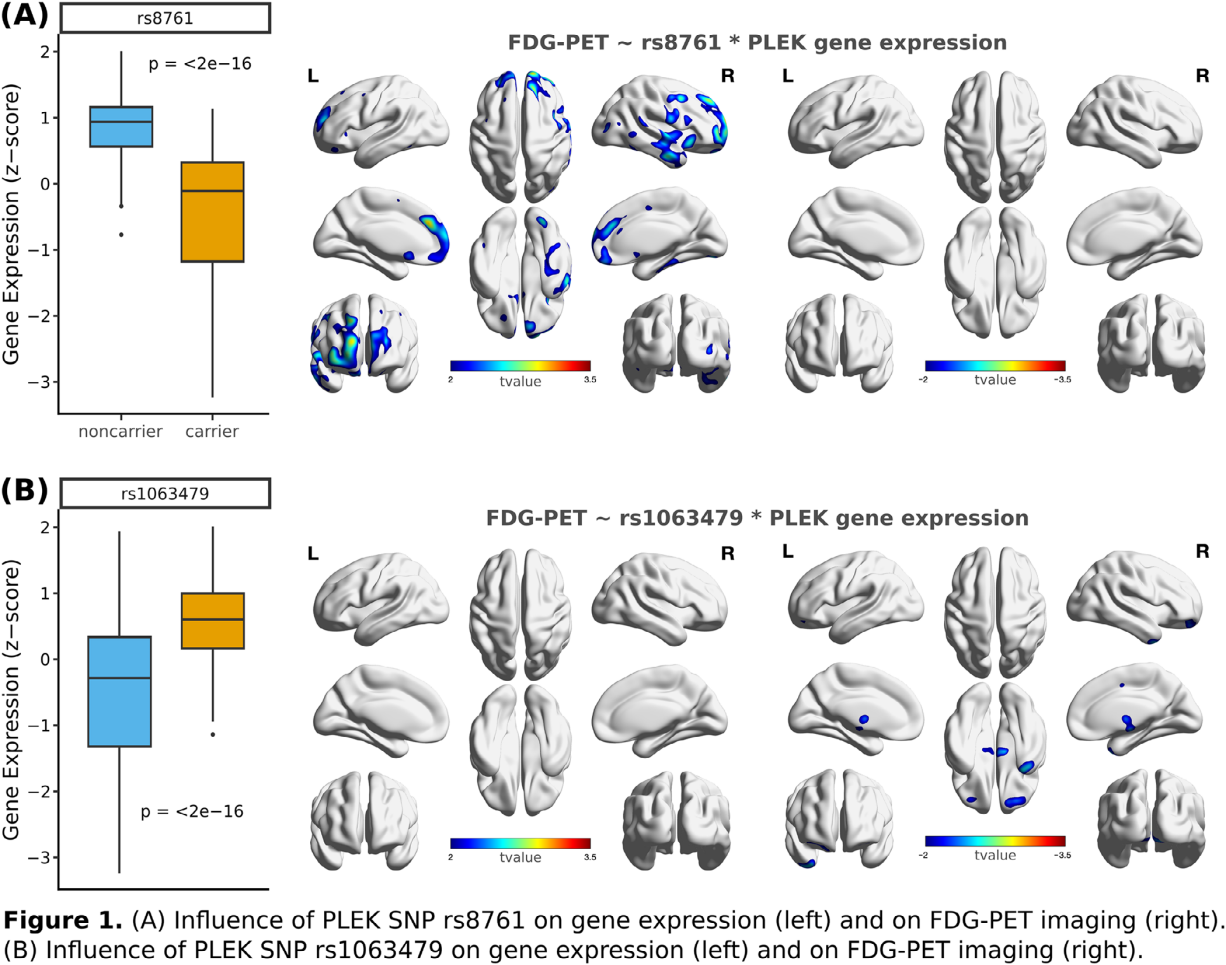# Influence of PLEK Genetic Variants on Brain Glucose Metabolism

**DOI:** 10.1002/alz70855_104281

**Published:** 2025-12-24

**Authors:** Marco De Bastiani, Christian Limberger, Débora Guerini de Souza, Bruna Bellaver, Guilherme Povala, Tharick A Pascoal, Pedro Rosa‐Neto, Eduardo R. Zimmer

**Affiliations:** ^1^ Universidade Federal do Rio Grande do Sul, Porto Alegre, Rio Grande do Sul, Brazil; ^2^ Brain Institute of Rio Grande Do Sul, PUCRS, Porto Alegre, RS, Brazil; ^3^ University of Pittsburgh, Pittsburgh, PA, USA; ^4^ Departments of Psychiatry and Neurology, University of Pittsburgh School of Medicine, Pittsburgh, PA, USA; ^5^ McGill University, Montreal, QC, Canada

## Abstract

**Background:**

Alzheimer's disease (AD) is a progressive neurodegenerative disorder shaped by genetic factors. Expression quantitative trait loci (eQTL) mapping identifies how genetic variants regulate gene expression, offering a powerful tool for uncovering the mechanisms underlying the disease. In this study, we adopted a data‐driven approach to explore the relationships between genetic variants, blood gene expression, and FDG‐PET imaging using the ADNI dataset. We hypothesized that regulatory effects revealed through eQTL mapping are associated with changes in brain FDG‐PET imaging.

**Methods:**

We analyzed genomic and blood transcriptomic data from 746 individuals across the AD continuum in the ADNI dataset. eQTL mapping of gene–single nucleotide polymorphism (SNP) pairs was performed using the MatrixEQTL package in the R statistical environment, accounting for age and sex covariates. Significance was defined as an FDR‐adjusted *p*‐value < 0.01 and an absolute beta value > 0.5. Subsequently, voxel‐wise regression analyses on FDG‐PET imaging data were conducted with the RMINC package to evaluate associations between gene–SNP interactions and brain glucose metabolism.

**Results:**

Out of 10,558 genes and 477,532 SNPs analyzed, 5,278 SNPs were significantly associated with changes in the expression of 217 genes. We analyzed the 11 SNP pairs with the strongest effects on expression patterns for each gene, including both upregulation and downregulation. Voxel‐wise regression analysis assessed the interaction between SNP carriership and gene expression on FDG‐PET. This revealed that carriers of the PLEK SNP rs8761, associated with PLEK gene downregulation (Figure 1A), exhibited FDG‐PET hypermetabolism in the frontal and temporal cortices. In contrast, the PLEK SNP rs1063479, linked to PLEK gene upregulation, did not show significant changes in the FDG‐PET signal (Figure 1B).

**Conclusion:**

The *PLEK* gene encodes pleckstrin, a plasma protein involved in the innate immune response and expressed in glial cells. Variants of *PLEK* associated with FDG‐PET imaging suggest a potential link between a plasma protein related to immune cells and brain glucose metabolism. To our knowledge, the role of *PLEK* in brain glucose metabolism has not been previously reported. Therefore, future studies should explore these associations to better understand the underlying mechanisms and their implications for AD pathology.